# Moving from conventional to adaptive risk stratification for oropharyngeal cancer

**DOI:** 10.3389/fonc.2024.1287010

**Published:** 2024-03-14

**Authors:** Vlad C. Sandulache, R. Parker Kirby, Stephen Y. Lai

**Affiliations:** ^1^ Bobby R. Alford Department of Otolaryngology- Head and Neck Surgery, Baylor College of Medicine, Houston, TX, United States; ^2^ Ear Nose and Throat Section (ENT), Operative Care Line, Michael E. DeBakey Veterans Affairs Medical Center, Houston, TX, United States; ^3^ Center for Translational Research on Inflammatory Diseases, Michael E. DeBakey Veterans Affairs Medical Center, Houston, TX, United States; ^4^ Department of Head and Neck Surgery, Division of Surgery, University of Texas MD Anderson Cancer Center, Houston, TX, United States; ^5^ Department of Molecular and Cellular Oncology, Division of Surgery, University of Texas MD Anderson Cancer Center, Houston, TX, United States; ^6^ Department of Radiation Oncology, Division of Surgery, University of Texas MD Anderson Cancer Center, Houston, TX, United States

**Keywords:** oropharynx, hyperpolarized MRI, circulating tumor cells, cell free DNA, smoking, radiation

## Abstract

Oropharyngeal cancer (OPC) poses a complex therapeutic dilemma for patients and oncologists alike, made worse by the epidemic increase in new cases associated with the oncogenic human papillomavirus (HPV). In a counterintuitive manner, the very thing which gives patients hope, the high response rate of HPV-associated OPC to conventional chemo-radiation strategies, has become one of the biggest challenges for the field as a whole. It has now become clear that for ~30-40% of patients, treatment intensity could be reduced without losing therapeutic efficacy, yet substantially diminishing the acute and lifelong morbidity resulting from conventional chemotherapy and radiation. At the same time, conventional approaches to de-escalation at a population (selected or unselected) level are hampered by a simple fact: we lack patient-specific information from individual tumors that can predict responsiveness. This results in a problematic tradeoff between the deleterious impact of de-escalation on patients with aggressive, treatment-refractory disease and the beneficial reduction in treatment-related morbidity for patients with treatment-responsive disease. True precision oncology approaches require a constant, iterative interrogation of solid tumors prior to and especially during cancer treatment in order to tailor treatment intensity to tumor biology. Whereas this approach can be deployed in hematologic diseases with some success, our ability to extend it to solid cancers with regional metastasis has been extremely limited in the curative intent setting. New developments in metabolic imaging and quantitative interrogation of circulating DNA, tumor exosomes and whole circulating tumor cells, however, provide renewed opportunities to adapt and individualize even conventional chemo-radiation strategies to diseases with highly variable biology such as OPC. In this review, we discuss opportunities to deploy developing technologies in the context of institutional and cooperative group clinical trials over the coming decade.

## Introduction

### OPC incidence is increasing

With more than 63,000 cases annually in the US, head and neck cancer (HNC) represents a significant health burden (1). The epidemic increase in human papillomavirus (HPV)-associated HNC will dramatically increase this burden over the coming decades. Given the epidemiologic shift toward HPV-mediated diseases and lagging vaccination rates, the rise in HNC incidence is projected to not *begin* to abate until the 2050s. The US healthcare system will need to accommodate medical conditions related to HNC and HNC treatment for well over 4 million patients projected to be diagnosed between 2000 and 2060 (2). Previously a relatively rare entity, HNC was primarily attributable to tobacco and alcohol exposure and was predominantly a disease of elderly male patients (3, 4). The rise in HPV-associated HNC (3, 5) affects most age groups and crosses gender and racial/ethnic barriers (6). Long known to be a cause of cervical, penile, and anal cancer (7–9), HPV has been shown to be the primary driver of the increase in HNC diagnoses particularly for the oropharynx site (OPC). Preclinical and clinical studies have now conclusively linked HPV to OPC tumorigenesis in a majority of new diagnoses in the United States, with an increasing incidence across much of the world (10–19).

### Survival is highly variable

The shift toward HPV-associated disease was accompanied by the first significant improvements in HNC treatment response and survival in the last 50 years of clinical research and medicine. First brought to light by the landmark retrospective analysis of RTOG 0129 by Ang et al. (10), HPV-associated OPC demonstrates a drastically improved survival compared to its HPV-independent counterpart. At a population level, younger OPC patients, without a history of tobacco exposure and early T-stage tumors were shown to have a significantly improved survival in the early 2000s compared to the previous half century (3). Despite these promising shifts in survival, the same analysis showed that a subset of OPC patients continues to demonstrate poor disease free and overall survival, consistent with historical data, despite application of new therapeutic strategies (3, 10).

Following a decade of clinical trial and retrospective data analysis, the AJCC Staging Manual received a significant update in its 8^th^ Edition, with a dichotomization of OPC into HPV-associated and HPV-independent disease, and a concomitant reduction in stage in the context of HPV-associated OPC meant to more accurately reflect the improved survival of patients with what in the past would have been considered Stage II-III and even Stage IV disease (20). The newest large scale clinical trials conducted in OPC, including RTOG1016 and De-ESCALaTE confirmed that the survival parameters for HPV-associated OPC had indeed shifted critically compared to historical data (21, 22).

This improvement in survival, predicated on an excellent response to conventional radiation and chemotherapy strategies in a large subset of HPV-associated OPC has given hope to patients and clinicians alike, given the prevailing failures to improve HNC survival over previous decades. Yet at the same time, the same improvement in survival has drastically complicated the clinical management of the disease, at a time when its increasing incidence is exacerbating potential errors in an exponential fashion. Although as a group, HPV-associated OPC patients do well compared to their HPV-independent OPC counterparts, this effect is not uniform. There remains considerable heterogeneity in HPV-associated OPC response to treatment. Among Veterans with high rates of heavy tobacco exposure, survival for HPV-associated OPC remains lower compared to non-smokers by approximately 20% (4, 23) in line with the Ang et al. intermediate-risk rates (4, 24). These characteristics are conserved in both white and black patients, resulting in similar disease behavior and oncologic outcomes (25). Re-analyzed data from RTOG 0129 and RTOG 0522 demonstrated that the overall (OS) and progression free survival (PFS) rates for low-, intermediate- and high-risk OPC patients persisted with a difference in PFS between low- and intermediate- risk groups of over 15% (26). Our recent analysis of over 600 OPC patients treated in the modern era showed that heavy tobacco exposure reduced survival by the same amount as a shift in disease stage of 1 (e.g., stage I migrated to stage II) (27), in line with data published earlier by Vawda et al. (28).

Whereas some risk factors (e.g., tobacco) portend inferior survival in a subset of HPV-associated OPC patients, there is increasing evidence that a subset of HPV-associated OPC patients demonstrates excellent response to chemo-radiation. A recent analysis of over 1000 HPV-associated OPC patients showed that low levels of multinucleation identified on analysis of pre-treatment biopsy specimens were associated with dramatic improvements in overall, disease free and distant metastasis free survival, with hazard ratios ranging from 1.78 to 1.94 (29). In parallel, even when the analysis is restricted by stage, as was done by our collaborative group in a cohort of 439 stage I patients, infiltrative lymphocyte levels can drive further stratification of survival with hazard ratios >2.0 (30).

Together these data indicate that new HPV-associated OPC patients cannot be expected to demonstrate uniform response to chemo-radiation and thus equivalent survival. Furthermore, there is no evidence that this divergent survival is likely to change over the coming decades due to significant shifts in treatment paradigms. Surgery has not replaced radiation for most patients and there is no evidence that post-treatment function will be better with surgery (31–33). Targeted agents are inferior to conventional chemotherapy and no less toxic (21, 22). Immunotherapy has not yet demonstrated utility in the definitive, frontline setting for HNC and thus will be unlikely to replace conventional chemotherapy as a radiosensitizer in the near future (34). The only viable option to achieve a precision oncology approach that appropriately balances treatment effectiveness and toxicity is to maximize separation of patients into high-risk and low-risk groups.

### Limitations to conventional risk stratification

Although largely self-evident, it remains important to understand why it is critical that we accurately risk-stratify OPC patients. Standard NCCN guidelines for OPC treatment include definitive external beam radiation (EBRT) regimens (66-70Gy) or surgical resection followed by adjuvant EBRT with a slight reduction in dose based on pathologic features of the disease and conventional chemotherapy with cisplatin being the current standard of care (21, 22). As indicated above, these conventional approaches are extremely effective in a majority of HPV-associated OPC patients but they carry significant acute toxicity and the potential for life-long debilitating morbidity (e.g., chronic renal insufficiency, peripheral neurotoxicity, chronic aspiration, lower cranial nerve neuropathies) (35–43). There is currently no definitive evidence that we can safely shift away from current NCCN guidelines for HPV-associated OPC disease as a whole. Omission of cisplatin has not been shown to be safe at a population level prospectively (HN002) (44) and direct replacement of cisplatin with cetuximab has failed in 2 prospective clinical trials (21, 22). Replacement of cisplatin with immune checkpoint inhibitors does not appear to be on the horizon for at least another decade based on the most recent negative clinical trial data (34). Altered fractionation regimens designed to reduce EBRT toxicity have been investigated for over 3 decades without a significant impact, although IMRT has indeed greatly reduced toxicity over previous EBRT delivery approaches (35). Dose de-escalation appears promising in very select patients, but has not yet been shown to be safe across the broader HPV-associated OPC population in large randomized clinical trials. Incorporation of surgery into treatment paradigms for OPC has shown promise as it relates to risk stratification and tailoring adjuvant treatment to disease burden. In EA3311, investigators were able to show that patients deemed intermediate-risk based on surgical pathologic parameters could receive a reduced dose of adjuvant radiation of 50Gy without a clear decrease in treatment efficacy as measured using progression free survival (PFS) (45).

Recurrence from HPV-associated OPC is deadly; no less so compared to that from HPV-independent disease. Salvage with surgery, re-irradiation or systemic treatment fails in >60% of recurrent disease patients (46–49). Taken together, the severe toxicity from current treatment regimens and the nearly uniform fatality of recurrent disease create a Hobson’s choice for patients and a difficult balancing act for oncologists. Reducing treatment intensity at a population level will undoubtedly result in more recurrences yet failure to reduce treatment intensity will result in overtreatment and unnecessary toxicity in a large fraction of the OPC population. Importantly, some of this toxicity will translate into treatment related mortality (e.g., aspiration), making the need for accurate risk-stratification of OPC patients critical.

Conventional risk stratification has been standard for HNC ever since the first introduction of TNM classification and has continued throughout the 8 editions of the AJCC Staging Manual. That conventional risk stratification is clinically useful is evidenced by the significant divergence of survival by disease stage across tens of thousands of treated patients; that incorporation of HPV status into OPC staging has been impactful is similarly made plain by both prospective and retrospective datasets (10, 11, 21, 27, 46, 50). Yet at the same time, conventional risk stratification has had a modest impact on our ability to develop treatment regimens better tailored to disease biology. Whereas positive margins and extra-nodal extension (ENE) were shown to be useful in assigning patients to treatment escalation with the addition of conventional chemotherapy in the adjuvant setting, their utility in the setting of HPV-associated disease may be more limited (47). For aggressive, advanced-stage disease, attempted escalation with induction chemotherapy failed to improve survival in the PARADIGM and DECIDE trials (51, 52), and in a recent in-depth retrospective analysis appeared to be associated with reduced survival in OPC patients (53). As mentioned above, changing from cisplatin to cetuximab, a drug assumed to be more tolerable and thus better suited for the lower risk HPV-associated OPC population failed to maintain adequate survival in both RTOG1016 (which included intermediate-risk OPC) and De-ESCALaTE (which included exclusively low-risk OPC) trials (21). HN002 concluded that although a modest reduction in EBRT dose was safe, the omission of cisplatin could not be deemed safe even in non-smokers with HPV-associated OPC (low-risk OPC) (44).

One limitation of conventional risk stratification is that it requires very large signals (difference in survival), very large cohorts or both. An excellent example of this is the initial Ang et al. study in which HPV-associated OPC demonstrated ~75% survival at 2 years compared to HPV-independent OPC patients which demonstrated ~30% survival at 2 years, with HPV-associated smokers essentially in the middle (10). These very large differences have persisted in retrospective analysis across multiple cohorts and are reproduced in the aggregate when data from RTOG1016 and De-ESCALaTE are analyzed head to head. Despite decades of investigation, no other biological variable in HNC has demonstrated such dramatic stratifying effects (e.g., *TP53*) across multiple prospective and retrospective cohorts and thus, no other biological variables are included in the AJCC staging or considered in NCCN guidelines for HNC treatment generally. Effect sizes from shifts in treatment are similarly small. When averaged over tens of thousands of patients, the effect size for adding conventional chemotherapy to radiation in the definitive setting results in merely a 7-8% improvement in survival in the latest MACH-NC analysis, yet its elimination in the setting of low risk disease has not been shown to be safe (54). For decades, nodal metastasis was considered one of the most compelling predictors of survival in HNC, and indeed for HPV-independent disease it remains so as was recently show in oral cavity disease (47). In contrast, HPV-associated OPC demonstrates excellent survival even when nodal metastasis is present, which resulted in the substantial down-staging of tumors with significant nodal disease in the 8^th^ edition of the AJCC Staging Manual (20).

A second limitation of conventional risk stratification is a fundamental lack of knowledge - we simply don’t know what we don’t know. Decades were required to properly observe the presence of, measure the impact for, and develop risk stratification based on, HPV status alone in OPC. More recent work from us and others has now identified other potential stratifiers for treatment response and survival, including multinucleation, infiltration of tumors by cytotoxic immunocytes, the presence of complex immune frameworks in a subset of OPC tumors and differential tumor mutational burden (30, 46, 55, 56). Yet none of these potential risk-stratification markers are fully proven, and it is unlikely that they would be incorporated into staging and used for treatment de-intensification without extensive prospective testing.

One critical limitation to incorporating biologically specific biomarkers into risk stratification algorithms stems from the potential for false negative findings. Many individual genomic events (e.g., *TP53* mutation, *KEAP1* mutation) can be quite rare depending on the subtype of OPC and thus most retrospective and prospective institutional datasets and even cooperative group trial cohorts will be underpowered to truly examine their risk stratification potential. The need to develop large cohorts, with comprehensive clinical data and appropriate matching tissue has now been recognized by investigators and funding agencies alike (e.g., National Institute of Dental and Craniofacial Research).

### Risk-stratification and therapeutic response drivers

Many aspects of tumor biology can confer “risk” as manifested by reduced survival. However, only those biological events which drive treatment response can really inform our ability to modulate existing therapeutic strategies in a meaningful way to reduce toxicity or improve overall response. In breast cancer and prostate cancer, hormonal receptor status is utilized to characterize the disease because it fundamentally influences response to hormonal blockade (57, 58). In melanoma and to a lesser degree in thyroid carcinoma, *BRAF* mutational status is a critical biomarker because it predicts response to a specific treatment, namely BRAF +/- MEK inhibition. Unlike in these diseases, and multiple other examples in adjacent solid tumors (e.g., lung cancer) (59, 60), HNC broadly and OPC in particular manifests few, if any, examples of biologically consistent drivers of response to chemotherapy and radiation which can be used to *mechanistically inform modulation of therapy, especially de-escalation strategies*.

Even within the context of HPV-driven disease, the superior response of disease to conventional chemotherapy and radiation remains unclear. Some speculate that maintenance of a wild-type *TP53* status allows for activation of the tumor suppressor under oxidative stress conditions (e.g., during treatment) and may explain the improved response rate (61). Others, including us, believe that an improved tumor immune micro-environment (i.e., enriched for functional immunocytes) may somehow result in an improved response, although this is somewhat mechanistically unclear since HPV-associated tumors do not demonstrate a substantially better response to immune checkpoint inhibitors compared to their HPV-independent counterparts (46, 62, 63). Another subset of investigators suggest that higher levels of oncogene-driven replication stress in HPV-associated tumors allows them to more easily activate programmed cell death pathways (61) or that non-canonical p16 signaling may be key to enhanced radiation response in this disease subset (64). The fact that we cannot consistently explain WHY HPV-associated OPC responds better to radiation (with or without chemotherapy) provides a clear impediment to a logical escalation or de-escalation strategy for this patient population. Whereas HPV oncogenic infections and their downstream impact on intra-cellular tumor suppressors and signaling cascades have been studied for years, some of the more recent pathomic and radiomic features correlated with improved survival in HPV-associated disease have never been mechanistically explored and thus are highly unlikely to really impact treatment intensity decisions for the near future without extensive preclinical and clinical investigation.

This limitation also applies to what many consider the treatment of the future, namely immunotherapy in the form of immune checkpoint inhibitors (ICIs). Starting with CheckMate141 and followed by Keynote048, ICIs have now demonstrated meaningful activity in HNC broadly and OPC specifically in the recurrent metastatic disease setting (63, 65). However, their use has encountered some of the same difficulties experienced when trying to improve upon the radiation vs surgery +/- conventional chemotherapy approach with targeted agents (e.g., cetuximab) or conventional induction chemotherapy in previous decades: treatment optimization. Combinatorial therapy studies have failed in the definitive upfront setting to date (e.g., JAVELIN Head and Neck 100) (34). In part, this is likely driven by the same limitation we face with conventional treatment. We have no predictive biomarker of ICI response in HNC or OPC specifically. PDL1 status although utilized, is far from being informative enough to further optimize utilization beyond the dichotomous chemotherapy versus no chemotherapy decision point. More sophisticated transcriptomic approaches published in recent years (e.g., TGEP) or our pathomic approaches (MuNI, OP-TIL) remain far from being prospectively validated and even with validation they remain poorly linked mechanistically to ICI effects (29, 30, 66, 67). It is also important to note, that immunotherapy in the form of existing ICIs, is not quite as benign as was initially hoped. Significant levels of immunotherapy-related adverse events (irAEs) have been reported in non-small cell lung cancer (NSCLC) (68, 69), melanoma (70) and HNSCC (71) especially when multiple ICIs are combined. Particularly problematic is the consistent observation that ICI toxicity and effectiveness are extremely correlated suggesting a substantial hurdle to ICI deployment for HNSCC particularly when combined with other toxic regimens/treatments.

### Adaptive risk stratification

In the second half of the last century, John Boyd introduced the OODA (observe, orient, decide, act) loop concept, first in the context of military conflict and then more generally in the context of human behavior and interaction. Conventional risk stratification for cancers has optimized the utilization of the OODA concept, even more so with the revolution in genomic, transcriptomic and proteomic characterization of tumors. However, a key component of Boyd’s approach to action was the loop itself, the iterative and ever informative nature of a repetitive cycle. Modern risk stratification runs the loop once; after the decision to act is made, no further information is easily available to the oncologist until the complete course of chemo-radiation runs its course. This approach violates basic principles of biology, which is adaptive in the setting of exogenous stress (particularly in highly flexible cancer cells) in addition to reducing the proven benefits of the loop. Oncologists are not to blame for this failure. The failure stems from the difficulties of obtaining new information from solid tumors that are meaningful, actionable, and timely. Yet new techniques are being increasingly deployed which may make this a reality in the not-too-distant future.

**Figure 1:**
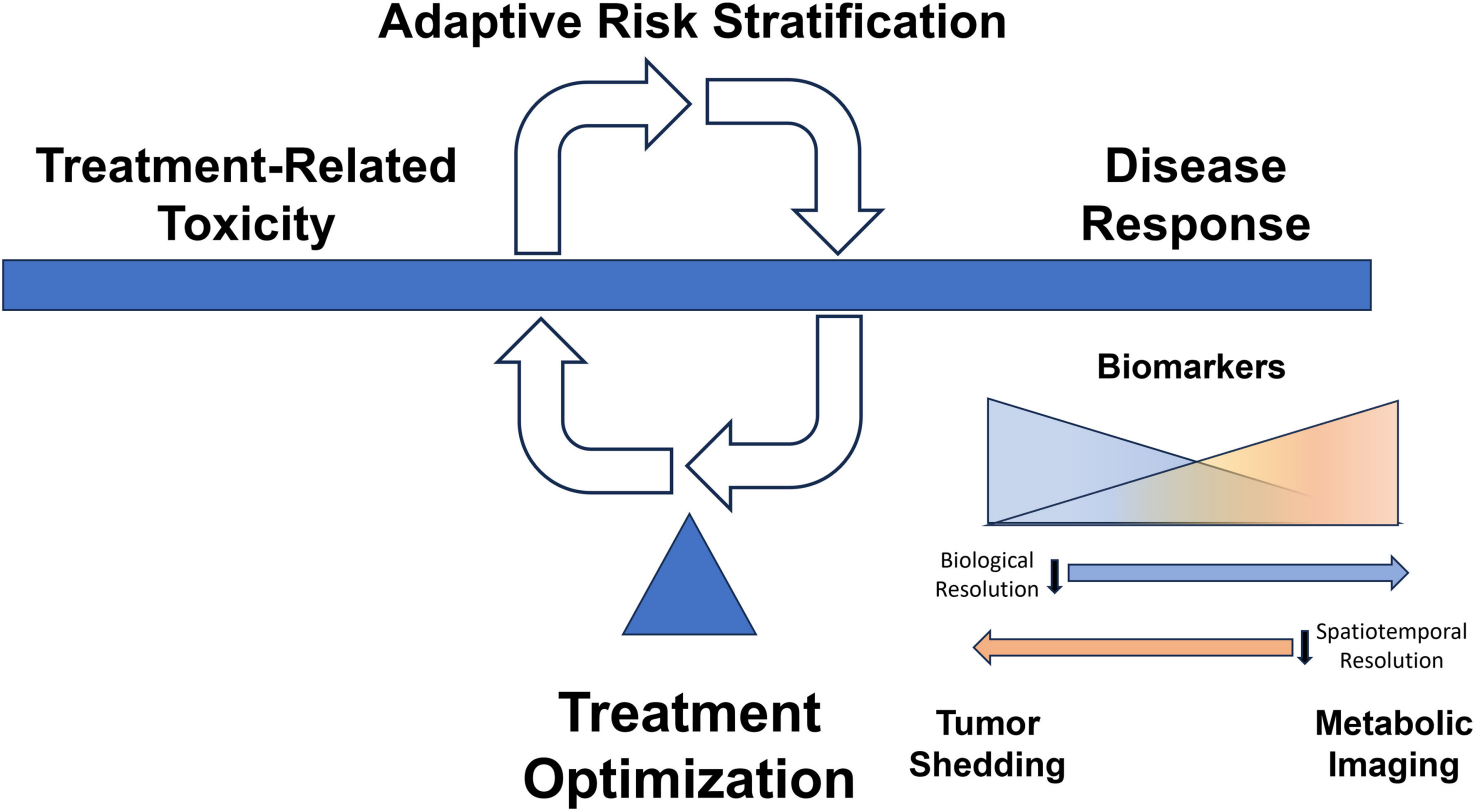
Dynamic Adaptive Risk Stratification. Treatment decisions for our patients balance maximizing disease response and minimizing treatment-related toxicity. There is currently a scarcity of clear biologically consistent drivers of response to therapy which can be used to mechanistically inform modulation of therapy, especially de-escalation strategies. Dynamic assessment of treatment response may allow therapeutic modification to balance disease control with toxicity. Tumor shedding creates a multitude of circulating biomarkers (e.g., viral DNA, tumor exosomes, viable circulating tumor cells) that provide high biological resolution regarding response to therapy, while imaging-based parameters may afford high spatiotemporal resolution reflective of tumor heterogeneity in response to treatment.

In addition to the difficulties associated with conventional risk stratification outlined above, conventional risk-stratification suffers from a fatal flaw. It is static; it ignores the effect of the treatment itself which can manifest in many ways. Radiation can impact ICI response through local destruction of immunocytes. Chemotherapy can impact ICI effectiveness through systemic myelosuppression. Both can generate significant shifts in tumor biology which may be anti-immunogenic (72). Conversely, these interactions can occur in a positive feedback loop through damage-associated molecular patterns (DAMPs) or generation of mutational or more commonly expression-based neoantigens (73, 74). Unlike other solid cancers, truly ingrained events such as *BRAF*, *ALK* and *EGFR* mutations simply do not exist in OPC or even HNC with sufficient frequency to drive treatment selection on the basis of predicted response. As a result, all biological shifts during treatment, small and subtle as they may be, can greatly impact the effectiveness of the chosen treatment and affect the predictive potential of any risk stratification schema (**Figure 1**). This limitation applies to ICIs as well which still lack a robustly informative biomarker of response in OPC and to some degree in many other solid tumors.

### Leveraging tumor shedding for adaptive risk stratification

While hematopoietic malignancies have an intrinsic circulating component, solid tumors are highly anatomically restricted and defined (even in the metastatic setting). However, the presence of solid tumors can be detected at a systemic level through a multitude of circulating markers, including viral DNA (for oncogenic viruses such as EBV and HPV), tumor exosomes, cell free DNA (cfDNA), and even fully viable circulating tumor cells. These markers provide a compelling avenue to indirectly interrogate events in solid tumors to inform treatment selection and make clinical decisions in an iterative fashion for an individual patient.

Plasma EBV DNA levels have been capable of detecting a prior infection and associated malignancies for over 2 decades (75, 76). Nearly 80% of patients with active nasopharyngeal cancer mediated by EBV shed detectable EBV DNA prior to treatment and EBV remains systemically detectable in the post-treatment setting when patients presented with initially higher stage disease (77). In contrast to serology, circulating DNA levels can be at least partially correlated to relative tumor burden generating a more useful biomarker of relative tumor burden in the post treatment setting (78). Recent studies have extended this approach to the HPV counterpart of EBV leveraging the fact that both are oncogenic viruses with a direct link to the biological genesis of the underlying disease. Oncogenic HPV infection can be detected at a single-cell level in basal keratinocytes suggestive of potential for a mechanistic biomarker with a high sensitivity albeit likely a low specificity for development of cancer in the short term (79). HPV viral loads have been correlated with survival in patients with OPC (80) in both retrospective and prospective series. The ability to detect measurable changes in circulating tumor tissue modified viral DNA (TTMV) during treatment holds some potential to inform de-escalation strategies for patients with HPV-associated OPC. Although the accuracy of such a biomarker would need to be extremely high, a more proximate application of this approach is as early biomarker of recurrence. TTMV has been utilized in large series (81) of patients (>1000) to track recurrence post-treatment with an overall positive predictive value for recurrent disease of 95% and a point-in-time negative predictive value is 95% (with the caveat that some patients with a one-time negative test did go on to develop recurrence). Detection of EBV and HPV can thus be useful but is not currently actionable as it does not reflect events downstream from the individual viral oncogenes and thus cannot inform how chemotherapy, radiation or ICIs might interact with an individual tumor’s biological features.

Whereas viral DNA can be useful in the setting of virally mediated HNC, circulating tumor DNA (ctDNA) can be broadly utilized regardless of underlying tumor pathogenesis. We and others have previously deployed ctDNA to detect actionable oncogenic events in solid tumors including melanoma and anaplastic thyroid carcinoma such as the V600E BRAF mutation (82). Other investigators (83) have used ctDNA and phylogenetic analysis to track the evolution of lung cancer and development of chemotherapy resistance. In contrast to HNC and NSCLC, in SCLC high rates of hematogenous spread are commonly encountered resulting in rapid and widespread distant metastasis. In this setting ctDNA is thought to be particularly informative and representative of the intrinsic tumor biology (84) as was shown via paired analysis of primary tumors and ctDNA of variant allele frequency of clonal mutations. Simply put, shifts in ctDNA during and post-treatment can reflect, albeit with caveats, similar shifts within the primary and metastatic tumor sites which may be indicative of cure or recurrence as a function of clonal expansion and/extinction. Recent work by Cao et al. highlighted the utility of a combined ctDNA/imaging-based approach to early detection of treatment response in AJCC (8^th^ edition) stage III OPC patients and demonstrate significant correlation with freedom from disease progression (85). Similarly, Chera et al. showed that rapid clearance of HPV ctDNA (defined as a favorable clearance profile) achieved cure with conventional chemo-radiation in contrast to patients with an unfavorable clearance profile (86).

A broader biological approach is to assess exosomes (87), sub-micrometer tumor cell vesicles, which can be stable in body fluids and contain not just DNA, but also RNA, tumor proteins, lipids, and metabolites. In some cases, proteins can be particularly informative as in the case of PDL1 (88) which has been correlated to HNC disease progression as compared to non-exosomal plasma PDL1 levels. Exosomes and their counterpart microvesicles (89) can be used in a largely agnostic fashion to characterize data from both tumor and viral DNA as well as associated proteins and metabolites, forming a biologically rich dataset and providing increased stability for macromolecules in inhospitable fluid environments such as saliva which can be of critical importance to HNC. At the extreme end of the spectrum, the entire biological landscape of a subset of tumor clones can be captured in the form of whole, viable circulating tumor cells (CTCs) (90). In HNC, a pooled survival analysis of 22 studies eligible for systematic review found that presence of CTCs was associated with shorter disease-free survival (DFS, HR 4.62, 95% CI 2.51-8.52) with a very high overall specificity but low sensitivity. An important limitation to circulating biomarkers is that their actionability remains in question at this time in the context of OPSCC. All existing systemic treatments inclusive of ICIs incur significant toxicity for limited survival benefit and almost none for lasting cure. As such, treatment in the recurrent/metastatic setting is reserved for either imaging identifiable lesions (e.g. radiation based treatment of oligometastasis, surgical resection of isolated regional recurrence) or for symptomatic disease (e.g. palliative intent chemotherapy and/or chemo-ICIs). Since there is limited evidence that earlier initiation of treatment is either feasible, in the setting of imaging invisible disease, or beneficial, in the setting of disseminated disease, the utility of early detection of recurrence/metastasis for this particular disease site remains unclear, particularly since it often precedes conventionally detectable disease by only several weeks to months. As such, utility may be initially limited to early detection of response to primary treatment that could assist escalation/de-escalation decision making.

### Leveraging metabolic imaging for adaptive risk stratification

Whereas ctDNA, CTCs and exosomes can provide high biologic resolution and identify a multitude of genomic, transcriptomic, and proteomic events related to tumorigenesis and evolution prior to and during treatment delivery, spatial resolution is absent. Although a signal may be detected, we have no idea where that signal is coming from (i.e., primary tumor, regional or distant metastases, etc). In contrast, imaging can provide outstanding spatial resolution, but significantly lower biological resolution. It is not the goal of this review to summarize the massive literature on the subject of biologic imaging of solid tumors, but rather to highlight some recent advances in imaging which may be applicable to dynamic or adaptive risk stratification strategies for OPC.

Starting with extensive work using F-labeled fluoromisonidazole (F-FMISO) (91), pre-treatment measurements of tumor hypoxia have long been utilized to ascertain potential radio-sensitivity/radio-resistance of whole tumors or individual tumor voxels given the known correlation between tumor hypoxia and radiation responsiveness. The counterpart of hypoxia, namely vascularity can be ascertained with fairly high sensitivity and specificity using dynamic contrast-enhanced MRI (DCE-MRI). DCE-MRI can be deployed in translationally relevant settings particularly when utilizing anti-angiogenic agents where imaging parameters may be altered prior to clinical effect (92). By capturing vascular parameters throughout the entire treatment field (tumor and adjacent normal tissue) DCE-MRI has the additional potential to be a real-time biomarker of normal tissue toxicity driven by shifts in vascularity. One such application pioneered by our group is the use of DCE-MRI for early detection of subclinical osteoradionecrosis (ORN) and identification of patients at high risk for severe ORN (93–95).

Extension of this work using multi-parametric (MRI) (96) has been used to predict complete response (CR) in patients with OPC prior to treatment completion in a manner suitable for potential treatment de-escalation in responders. Although additional work will be required to optimize multi-parametric and even DCE-MRI to fully capture biological data from the primary tumor and associated cervical lymphadenopathy common to OPC, preliminary findings are promising (97). This is particularly true since the approach appears to be scalable across institutions as shown in a comprehensive analysis (98) of the accuracy of diffusion-weighted imaging (DWI) for predicting locoregional failure of chemo-radiation in HNC across 9 studies and 421 patients, with a sensitivity of 82%, specificity of 70% and an area under the sROC curve of 84%.

While tumor vascularity, cellularity and hypoxia are transient on a slow scale (days-weeks), tumor metabolism is a continuously changing biological variable that has extremely high temporal resolution (minutes-hours), and when interrogated via metabolic imaging can be analyzed with an equally high spatial resolution. Over the last 2 decades, both FDG-PET and hyperpolarized magnetic resonance imaging (HP-MRI) techniques have been used to assess the aggressiveness of solid tumors including HNC and have been explored as tools to predict treatment response in preclinical models and patients (99–109). Although FDG-PET is available in clinical settings, prospective clinical trial data suggest that measurement of mid-therapy glucose uptake does not allow for adaptive reduction in tumor volumes (110–115). Furthermore, glucose uptake does not correlate with radiation response and provides no information on intracellular metabolic fluxes (116–118). In contrast HP-MRI of labeled pyruvate and lactate provides a unique opportunity to obtain real-time metabolic information from within solid tumors. Its ability to detect differential metabolic activity in tumor tissue has been established (99, 109). Substantial work from other groups has advanced the development of HP-MRI into a clinically viable tool for characterization of intrinsic tumor aggressiveness (prostate) and towards deployment of HP-MRI as a tool to measure treatment response (e.g., breast cancer) (101, 103, 119–121). HNC sensitivity to genotoxic agents is a function of multiple discrete biological events, such as activation of pathways associated with the human papillomavirus (HPV) or mutation of tumor suppressors such as *TP53.* Unfortunately, we and others have shown that individual patient responses are not completely uniform across patient groups (e.g., HPV-associated vs. HPV-independent, wildtype vs. mutant *TP53*), and this may be due in large part to the heterogenous activation of acquired resistance pathways once treatment starts (3, 4, 10, 122–127). Therefore, even if genomic biomarkers such as *TP53* and HPV start to be used in treatment-selection decisions at baseline, tailoring treatment intensity to *individual* patients in the face of acquired resistance potentially based upon changes in metabolic response will still be required for true precision oncology approaches and personalized cancer treatment.

In 2014, we were the first to show that *k_PL_* measured with noninvasive HP [1- (13)C]-pyruvate MRI is decreased under conditions of depleted REDOX following genotoxic stress in animal models of HNC and other tumors (128). We have developed a multi-compartment model of intracellular *k_PL_* which increases the fidelity of our measurements (129). In 2020, for the first time, we measured these metabolic changes in a patient during treatment. This first-in-human assessment of metabolic response to treatment serves as a critical proof-of-principle and demonstrates our technical capability to execute the proposed studies. On the basis of these robust preliminary data, we propose to test the potential of metabolic interrogation as a clinical tool that can (1) predict treatment response and (2) be used to develop treatment strategies tailored to individual tumor biology. Our innovative approach is supported by (1) studies that link reducing potential to genotoxic stress (127, 130–136); (2) clinical and preclinical data that link lactate to tumor progression and treatment response (137–139); and (3) studies that confirm the excellent spatiotemporal resolution of HP [1- (13)C]-pyruvate MRI (128, 140–143).

The biologically rich data from anatomic and metabolic studies can be enhanced by nearly an order of magnitude when combined with artificial intelligence (AI)/machine learning (ML) approaches. Utilization of machine learning approaches (144) can generate meaningful data even from relatively data poor CECT studies to identify radiomic features which when combined can distinguish invasive cancer from more benign solid tumors. This approach has also been deployed (145) to generate combined radiomic risk scores which can predict disease free and overall survival in the context of either conventional treatment or in the presence of immunomodulatory combinatorial strategies.

### A path toward clinical translation

Conventional and adaptive risk stratification are not mutually exclusive. They represent 2 aspects of a combined approach designed to deliver maximal anti-tumor activity, using the most appropriate agents, at the lowest possible dose that will achieve a durable cure. In order to maximize the therapeutic index of both conventional and targeted strategies the most effective future algorithms will start with conventional risk stratification that combines biological data with clinical risk factors. Upon this baseline approach, treatment algorithms will then incorporate a complex adaptive risk stratification strategy that combines feasible aspects of biological interrogation using circulating and imaging tumor markers (**Figure 1**). Critically, this second layer of data will be truly personalized, specific not only to the individual tumor, but also to the interaction between the individual tumor and the chosen treatment regimen. Successful implementation of such an approach will require a rigorous process, outlined by Pepe et al. nearly 2 decades ago (146), whose key ingredients include carefully defining the target population (carefully selected based on clinically relevant criteria and relevant disease biology) and the expected outcome for each individual biomarker (e.g. impact on local recurrence vs distant metastasis rates), testing in populations large enough to reduce the number of false negative studies, and *a priori* definitions of expected effect size and clinical impact. For solid tumors, which present challenges to repetitive interrogation with high biological and spatial resolution (see above), an “n of 1” precision oncology algorithm is somewhat unlikely using existing approaches and technologies, however, careful integrated of layered biomarkers can provide a significant advantage over current clinical paradigms for OPC. For widespread clinical translation it is critical to identify circulating markers (high biological resolution) and imaging modalities (high temporal and spatial resolution) which can be rapidly deployed and relatively cost-effective. Finally, the entire platform and associated algorithms must be readily replicated across institutions and healthcare delivery systems. Our patients deserve no less.

## Author contributions

SL: Conceptualization, Formal analysis, Funding acquisition, Project administration, Supervision, Writing – original draft, Writing – review & editing. VS: Conceptualization, Formal analysis, Funding acquisition, Project administration, Supervision, Writing – original draft, Writing – review & editing. RK: Writing – original draft, Writing – review & editing.
